# Voxel-based morphometry focusing on medial temporal lobe structures has a limited capability to detect amyloid β, an Alzheimer’s disease pathology

**DOI:** 10.18632/aging.104012

**Published:** 2020-10-05

**Authors:** Masashi Kameyama, Kenji Ishibashi, Jun Toyohara, Kei Wagatsuma, Yumi, Umeda-Kameyama, Keigo Shimoji, Kazutomi Kanemaru, Shigeo Murayama, Sumito Ogawa, Aya M. Tokumaru, Kenji Ishii

**Affiliations:** 1 Department of Diagnostic Radiology, Tokyo Metropolitan Geriatric Hospital and Institute of Gerontology, Tokyo 173-0015, Japan; 2 Research Team for Neuroimaging, Tokyo Metropolitan Institute of Gerontology, Tokyo 173-0015, Japan; 3 Department of Geriatric Medicine, The University of Tokyo School of Medicine, Tokyo 113-8655, Japan; 4 Department of Neurology, Tokyo Metropolitan Geriatric Hospital and Institute of Gerontology, Tokyo, 113-0015, Japan

**Keywords:** beta-amyloid, hippocampus, magnetic resonance imaging (MRI), Pittsburgh Compound B (PiB), positron emission tomography (PET)

## Abstract

Voxel-based morphometry (VBM) analysis of nuclear Magnetic Resonance Imaging (MRI) data allows the identification of medial temporal lobe (MTL) atrophy and is widely used to assist the diagnosis of Alzheimer’s disease (AD). However, its reliability in the clinical environment has not yet been confirmed. To determine the credibility of VBM, amyloid positron emission tomography (PET) and VBM studies were compared retrospectively. Patients who underwent Pittsburgh Compound B (PiB) PET were retrospectively recruited. Ninety-seven patients were found to be amyloid negative and 116 were amyloid positive. MTL atrophy in the PiB positive group, as quantified by thin sliced 3D MRI and VBM software, was significantly more severe (p =0.0039) than in the PiB negative group. However, data histogram showed a vast overlap between the two groups. The area under the ROC curve (AUC) was 0.646. MMSE scores of patients in the amyloid negative and positive groups were also significantly different (*p* = 0.0028), and the AUC was 0.672. Thus, MTL atrophy could not reliably differentiate between amyloid positive and negative patients in a clinical setting, possibly due to the wide array of dementia-type diseases that exist other than AD.

## INTRODUCTION

Voxel-based morphometry (VBM) is widely used to help diagnose Alzheimer’s disease (AD). It is a convenient tool that has gained importance due to the increasing accessibility of nuclear Magnetic Resonance Imaging (MRI). VBM normalizes the 3D MRI data of a subject to a standardized space, extracts gray matter data by segmentation, undertakes spatial smoothing, and statistically analyzes the data using a normal database. VBM is able to detect regions that are atrophic relative to the entire cerebral cortex. VBM software, such as Voxel-Based Specific Regional Analysis System for Alzheimer’s Disease (VSRAD ^®^, Eisai Co., Tokyo, Japan), provides a score by which medial temporal lobe (MTL) atrophy can be assessed objectively, thus, bypassing the need of specially trained staff for interpretation [1, 2].

AD has two main pathological features, senile plaques made of the β-amyloid (Aβ), which invariably occur as a part of the pathological process of AD, and neurofibrillary tangles (NFT) made of phosphorylated tau. NFT develops first in the MTL [3] causing atrophy. The MTL including the hippocampus and entorhinal and perirhinal cortices plays a very important role in memory [4]. Therefore, it is plausible that MTL atrophy might be a useful marker for detecting AD pathology. Several studies have reported that MTL atrophy can be detected in patients with AD from a very early stage [5–8] and, therefore, is useful to distinguish prodromal AD from normal aging [1, 9–11]. Particularly, the CA1 region of the hippocampus shows the most severe atrophy in AD [12–16]. Hippocampal volume provides a quantitative marker of the pathologic substrate that produces the observed cognitive deficit in AD [17].

Although VBM-derived MTL atrophy scores are easy to interpret, they are not without fault and can sometimes produce false positives, categorizing cognitively normal subjects as AD patients. General practitioners in Japan often refer healthy patients with high VBM scores to dementia specialists and prescribe dementia drugs such as donepezil (Aricept ^®^, Eisai Co., Tokyo, Japan) without undertaking memory examinations. This overprescribing of AD medications and unnecessary referral to dementia specialists places an extraneous burden on the Japanese health insurance system and medical infrastructure.

**Figure 1:**
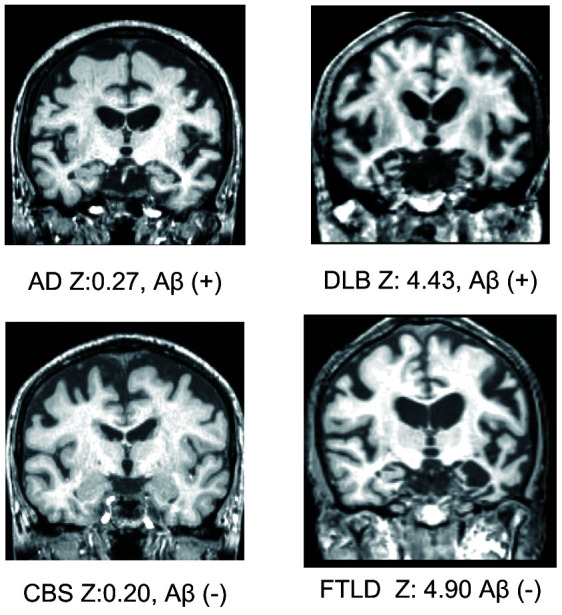
**Representative medial temporal lobe images, VSRAD *Z* scores, and amyloid positivity.**

Although a previous study showed that MTL atrophy scores calculated using VSRAD ^®^ Advance have a high sensitivity (86.4%) and a high specificity (97.5%) [2], there are two serious limitations of the study, which impede its reliability in an actual clinical setting. First, the study population included only AD and cognitively normal subjects. In reality, however, clinicians must be able to differentiate between the different types of cognitive disorders such as dementia with Lewy bodies (DLB), fronto-temporal lobe dementia (FTLD), progressive supranuclear palsy (PSP), cortico-basal degeneration (CBD), neurofibrillary tangle-predominant dementia (NFTD), and argyrophilic grain dementia (AGD). Representative MTL images are shown in Figure 1. A previous study showed that only 34% of patients with neurodegenerative dementia (clinical dementia rating scale; CDR ≥ 1) had AD pathology [18] and another showed that there was no significant difference in MTL atrophy between subjects with AD and non-AD dementia [17]. Therefore, while VSRAD ^®^ Advance may be useful for differentiating AD from cognitive normal subjects, the score alone is not sufficient to diagnose AD. Second, the patients assigned to the AD arm of the study were diagnosed based on clinical criteria. However, false positive diagnosis of AD is possible when using clinical criteria alone and similarly, patients with AD pathologies are often misdiagnosed with normal cognition [19]. A paper reviewing the reliability and validity of NINDS-ARDA Alzheimer’s criteria [20] found that the sensitivity and specificity of the ‘probable AD’ category was 76.6 – 70.9% and 59.5 – 70.8%, respectively; and those of the ‘probable AD’ and ‘possible AD’ categories combined were 87.3 – 82.7% and 44.3 – 54.5%, respectively [21]. Lim et al. [22] also showed that the ‘probable AD’ category had 83% sensitivity and 55% specificity; ‘probable AD’ and ‘possible AD’ categories combined had 85% sensitivity and 50% specificity. In a population-based study by Petrovitch et al. [23], only 65% of the clinically diagnosed AD was reported to be pathologically accurate. Amyloid imaging is reported to alter the presumptive diagnosis in approximately 30% of cases, increase the diagnostic confidence in about 60% of cases, and change the patient management in about 60% of cases [24]. Owing to the high reliability of amyloid positron emission tomography (PET), a positive result can be considered as a clear confirmation of AD pathology.

This study aimed to investigate the clinical reliability of VBM in diagnosing AD. We compared hippocampal atrophy assessed using VSRAD *Z*-score and amyloid PET retrospectively.

## RESULTS

### Demographics

Seventy-three out of 286 patients were excluded from the analysis. Eighteen patients did not have 3D MRI data suitable for VBM. MR images of 46 patients showed bad segmentation, two images were of low quality due to the head movement, and one showed susceptible artifact due to a cochlea implant. One patient had a large arachnoid cyst, one had a large infarction, and one had normal pressure hydrocephalus. Three patients with cerebral amyloid angiopathy were also excluded, as they may show amyloid positivity in the absence of senile plaques and MRI can be influenced by hemorrhage. Of the 213 remaining patients, 97 were amyloid negative and 116 were amyloid positive. Patient characteristics are shown in Table 1. For all patients, the diagnosis at the time of scanning and the most recent diagnosis by neurology specialists are shown in Supplementary Figure 1.

**Table 1:** Demographics.

	**Aβ (−)**	**Aβ (+)**	***p***
*n* (male/female)	97 (42/55)	116 (40/76)	0.188
age	71.8±10.2	72.7±10.0	0.515
age range	43 – 88	48 – 97	
MMSE*	24.0±6.8	20.6±7.5	0.003

± denotes standard deviation. *MMSE were available for 153 patients. χ^2^-test for sex ratio and *t*-test (bilateral) for age and MMSE were executed.

### Distribution of hippocampal atrophy

**Figure 2:**
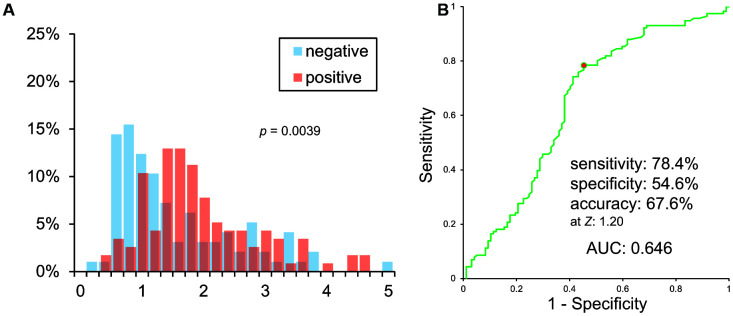
**(A) Histogram of VSRAD *Z* scores. VSRAD *Z* scores of PiB negative and positive patients were significantly different. However, histogram showed a vast overlap of scores between PiB positive and negative patients. (B) ROC analysis of VSRAD *Z* values.**

VSRAD *Z* scores of PiB negative and positive patients were significantly different (*p* = 0.00393). However, histograms showed a vast overlap of scores between the two groups (Figure 2A). A Receiver Operating Characteristic (ROC) curve of VSRAD *Z* values is shown in Figure 2B. VSRAD ^®^ had 78.4% sensitivity, 54.6% specificity, and 67.6% accuracy at a *Z* value of 1.20. The Area Under the ROC curve (AUC) was 0.646.

### MMSE

**Figure 3:**
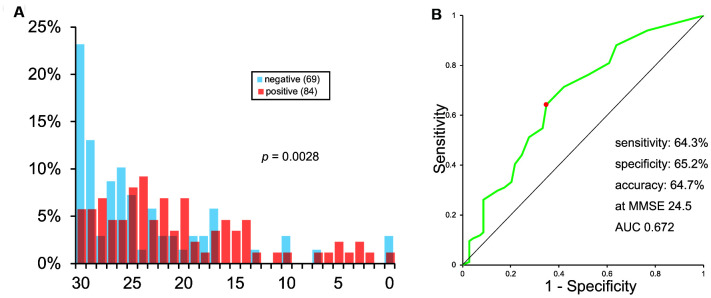
**Histogram (A) and ROC analysis (B) of MMSE scores.**

The histogram of MMSE scores is shown in Figure 3A. It also showed a vast overlap of scores between the two groups.

The ROC curve of MMSE scores is shown in Figure 3B. As calculated, the sensitivity, specificity, and accuracy of MMSE was 64.3%, 65.2%, and 64.7%, respectively, at MMSE 24.5. AUC was 0.672.

### Correlation analysis

We assessed the correlation between amyloid positivity and MMSE score, VSRAD *Z* score, age, and sex. The model was significant (χ^2^ = 23.42, *p* = 0.0029). The lack-of-fit test by logistic regression analysis showed that amyloid positivity was significantly correlated with the MMSE score (χ^2^ = 4.97, *p* = 0.0258), but not with the VSRAD *Z* score (χ^2^ = 1.44, *p* = 0.2301). Moreover, age (χ^2^ = 0.61, *p* = 0.4366) and sex (χ^2^ = 0.59, *p* = 0.4428) were not significantly correlated with amyloid positivity. This indicates that MMSE has a closer correlation with amyloid positivity than VSRAD *Z* scores.

## DISCUSSION

We observed that MTL atrophy, determined using VBM with MRI was not a reliable indicator of Aβ deposition. The significant difference between the VSRAD *Z* scores of Aβ positive and negative patients can be explained by a difference in MMSE score. The ROC analysis of VSRAD *Z* scores showed a low AUC value (0.646), which was similar to the AUC value of MMSE scores (0.672). Since MMSE is much simpler and inexpensive than MRI examination, MMSE would be preferable to VBM as a tool to diagnose AD.

The low reliability of VBM observed in this study is likely attributable to the existence of many different forms of dementias other than AD, a factor not accounted for in the previous study on VBM [2]. Although MTL atrophy detected by MRI correlates with NFT pathology, it is not specific to AD [17]. Furthermore, hippocampal-sparing AD, which does not involve hippocampal atrophy, is reported in approximately 11% of cases, which should also be taken into consideration when designing a study involving AD patients [25]. Early stage AD before hippocampal shrinkage [26] (preclinical stages of AD or MCI due to AD) would also result in a false negative VBM.

VBM for MTL atrophy and PiB PET for Aβ deposition target two different components of AD pathology, NFT and senile plaques, respectively. Tateno et al. [27] reported no correlation between Aβ deposition and MTL atrophy, while Jack et al. [28] reported a weak correlation. Studies showed that Aβ deposition in the neocortex is related to MTL atrophy only at a very early stage [29–31], and moreover the relationship is of a weak and inconsistent nature [32]. This phenomenon can be explained by the fact that Aβ accumulation reaches a plateau very early during the disease progression [26, 28]. Within Aβ (+) patients, hippocampal atrophy showed a significant correlation with Braak and Braak staging and the level of tau in the cerebrospinal fluid (CSF), moreover, hippocampal atrophy showed a weak correlation with Aβ burden [33].

Although our study demonstrated that VBM is not useful in diagnosing AD, it may be useful in other situations. VBM can be used to access MTL atrophy for research purpose [34]. It has been reported that the pattern of gray matter atrophy is associated with NFT pathology in Braak stage V and VI patients [35]. Identification of the atrophy pattern would be useful for classifying patients into the pathological subtypes of AD, *i.e.* typical AD, hippocampal-sparing AD, and limbic-predominant AD [36], and to distinguish nonAD degenerative dementia from MCI due to AD [37, 38]. Moreover, VBM is routinely used to evaluate disease-specific atrophic regions [39].

There are several limitations to this study. First, it should be noted that amyloid positivity does not conclusively equate to a diagnosis of AD, although an amyloid negative result can rule out the possibility of AD. Moreover, it takes many years to develop hippocampal atrophy after amyloid deposition [26]. Second, although the study population was large, this retrospective study might be biased since patients who are difficult to be diagnosed require amyloid PET scanning. Therefore, the patient population in this study may not be a true representation of the wider population. However, a pathological study showed that the proportion of patients with cognitive impairment with pure Alzheimer’s disease was as little as 34% [18]. Third, the MRI machine was updated to a newer model during the studied period. The difference in machines may influence the VBM results obtained. However, the effect is likely to be insignificant in clinical settings.

In conclusion, our study demonstrated that VBM based analysis of MRI data reliably detects hippocampal atrophy, but is not useful for the diagnosis of AD.

## MATERIALS AND METHODS

### Patients

286 patients, who underwent Pittsburgh Compound B (PiB) PET between March 2, 2006 and January 25, 2017, were retrospectively recruited. For patients who underwent PiB PET no less than twice during this period, the first scan was used.

### Ethical approval and consent to participate

The study was conducted in accordance with the Ethical Guidelines for Medical and Health Research Involving Human Subjects in Japan and conformed to the Helsinki Declaration. The study protocol was approved by the institutional review board of the Tokyo Metropolitan Institute of Gerontology. Patients and their families were provided with detailed information, and written informed consents were obtained from all participants.

### Amyloid PET

555 MBq of [^11^C] PiB was administered intravenously. Patients underwent either a 70 minute dynamic scan or a 20 minute static scan 40 or 50 minutes after administration of the radiotracer using PET scanner. Discovery PET/CT 710 (GE Healthcare, Waukesha, WI, USA) and Headtome V (Shimadzu Corporation, Kyoto, Japan) were the two machines used.

Amyloid positivity was determined by two experts (MK, K.Ishibashi) according to the standard criteria. Images in which the tracer accumulation was higher in the cortex or striatum than in the white matter were considered amyloid positive. Where the opinions of the two experts differed, categorization was determined through discussion.

### MRI

MR images for VBM (3D T_1_-weighted images) were executed on Signa Excite HD (1.5T), Signa HDxt (1.5T) or Discovery 750w (3T) (GE Healthcare, Waukesha, WI, USA). A VBM program based on Statistical Parametric Mapping (SPM) 8 with Diffeomorphic Anatomic Registration Through Exponentiated Lie algebra (DARTEL) (VSRAD ^®^ Advance 2, Eisai Co., Tokyo, Japan) [2] was applied and a VSRAD *Z* score, representing MTL atrophy, was obtained.

Segmentation error was assessed by two experts (MK, K.Ishibashi). Again, where the opinions of the two experts differed, a consensus was reached through discussion.

### Statistical analysis

All statistical analyses, excluding logistic regression analysis, were performed using a standard spread sheet software, Excel ^®^ 2016, (Microsoft Corporation, Redmond, WA, USA). Logistic regression analysis was performed using JMP ^®^ version 11.0.0 (SAS Institute Inc., Cary, NC, USA).

## Supplementary Material

Supplementary Figure 1

## References

[1] Hirata Y, Matsuda H, Nemoto K, Ohnishi T, Hirao K, Yamashita F, Asada T, Iwabuchi S, Samejima H. Voxel-based morphometry to discriminate early Alzheimer’s disease from controls. Neurosci Lett. 2005; 382:269–74. 10.1016/j.neulet.2005.03.038 15925102

[2] Matsuda H, Mizumura S, Nemoto K, Yamashita F, Imabayashi E, Sato N, Asada T. Automatic voxel-based morphometry of structural MRI by SPM8 plus diffeomorphic anatomic registration through exponentiated lie algebra improves the diagnosis of probable Alzheimer Disease. AJNR Am J Neuroradiol. 2012; 33:1109–14. 10.3174/ajnr.A2935 22300935 PMC8013247

[3] Braak H, Braak E. Neuropathological stageing of Alzheimer-related changes. Acta Neuropathol. 1991; 82:239–59. 10.1007/BF00308809 1759558

[4] Miyashita Y, Kameyama M, Hasegawa I, Fukushima T. Consolidation of visual associative long-term memory in the temporal cortex of primates. Neurobiol Learn Mem. 1998; 70:197–211. 10.1006/nlme.1998.3848 9753597

[5] Jack CR Jr, Petersen RC, O’Brien PC, Tangalos EG. MR-based hippocampal volumetry in the diagnosis of Alzheimer’s disease. Neurology. 1992; 42:183–88. 10.1212/wnl.42.1.183 1734300

[6] Jack CR Jr, Petersen RC, Xu YC, Waring SC, O’Brien PC, Tangalos EG, Smith GE, Ivnik RJ, Kokmen E. Medial temporal atrophy on MRI in normal aging and very mild Alzheimer’s disease. Neurology. 1997; 49:786–94. 10.1212/wnl.49.3.786 9305341 PMC2730601

[7] Karas GB, Scheltens P, Rombouts SA, Visser PJ, van Schijndel RA, Fox NC, Barkhof F. Global and local gray matter loss in mild cognitive impairment and Alzheimer’s disease. Neuroimage. 2004; 23:708–16. 10.1016/j.neuroimage.2004.07.006 15488420

[8] Whitwell JL, Przybelski SA, Weigand SD, Knopman DS, Boeve BF, Petersen RC, Jack CR Jr. 3D maps from multiple MRI illustrate changing atrophy patterns as subjects progress from mild cognitive impairment to Alzheimer’s disease. Brain. 2007; 130:1777–86. 10.1093/brain/awm112 17533169 PMC2752411

[9] Jack CR Jr, Petersen RC, Xu YC, O’Brien PC, Smith GE, Ivnik RJ, Boeve BF, Waring SC, Tangalos EG, Kokmen E. Prediction of AD with MRI-based hippocampal volume in mild cognitive impairment. Neurology. 1999; 52:1397–403. 10.1212/wnl.52.7.1397 10227624 PMC2730146

[10] Chételat G, Landeau B, Eustache F, Mézenge F, Viader F, de la Sayette V, Desgranges B, Baron JC. Using voxel-based morphometry to map the structural changes associated with rapid conversion in MCI: a longitudinal MRI study. Neuroimage. 2005; 27:934–46. 10.1016/j.neuroimage.2005.05.015 15979341

[11] Apostolova LG, Dutton RA, Dinov ID, Hayashi KM, Toga AW, Cummings JL, Thompson PM. Conversion of mild cognitive impairment to Alzheimer disease predicted by hippocampal atrophy maps. Arch Neurol. 2006; 63:693–99. 10.1001/archneur.63.5.693 16682538

[12] Csernansky JG, Wang L, Joshi S, Miller JP, Gado M, Kido D, McKeel D, Morris JC, Miller MI. Early DAT is distinguished from aging by high-dimensional mapping of the hippocampus. Dementia of the Alzheimer type. Neurology. 2000; 55:1636–43. 10.1212/wnl.55.11.1636 11113216

[13] Apostolova LG, Dinov ID, Dutton RA, Hayashi KM, Toga AW, Cummings JL, Thompson PM. 3D comparison of hippocampal atrophy in amnestic mild cognitive impairment and Alzheimer's disease. Brain. 2006; 129:2867–73. 10.1093/brain/awl274 17018552

[14] Chételat G, Fouquet M, Kalpouzos G, Denghien I, De la Sayette V, Viader F, Mézenge F, Landeau B, Baron JC, Eustache F, Desgranges B. Three-dimensional surface mapping of hippocampal atrophy progression from MCI to AD and over normal aging as assessed using voxel-based morphometry. Neuropsychologia. 2008; 46:1721–31. 10.1016/j.neuropsychologia.2007.11.037 18289618

[15] Frisoni GB, Ganzola R, Canu E, Rüb U, Pizzini FB, Alessandrini F, Zoccatelli G, Beltramello A, Caltagirone C, Thompson PM. Mapping local hippocampal changes in Alzheimer’s disease and normal ageing with MRI at 3 tesla. Brain. 2008; 131:3266–76. 10.1093/brain/awn280 18988639

[16] Apostolova LG, Mosconi L, Thompson PM, Green AE, Hwang KS, Ramirez A, Mistur R, Tsui WH, de Leon MJ. Subregional hippocampal atrophy predicts Alzheimer’s dementia in the cognitively normal. Neurobiol Aging. 2010; 31:1077–88. 10.1016/j.neurobiolaging.2008.08.008 18814937 PMC2873083

[17] Jack CR Jr, Dickson DW, Parisi JE, Xu YC, Cha RH, O’Brien PC, Edland SD, Smith GE, Boeve BF, Tangalos EG, Kokmen E, Petersen RC. Antemortem MRI findings correlate with hippocampal neuropathology in typical aging and dementia. Neurology. 2002; 58:750–57. 10.1212/wnl.58.5.750 11889239 PMC2745935

[18] Saito Y, Murayama S. Neuropathology of mild cognitive impairment. Neuropathology. 2007; 27:578–84. 10.1111/j.1440-1789.2007.00806.x 18021380

[19] Dubois B, Feldman HH, Jacova C, Dekosky ST, Barberger-Gateau P, Cummings J, Delacourte A, Galasko D, Gauthier S, Jicha G, Meguro K, O’Brien J, Pasquier F, et al. Research criteria for the diagnosis of Alzheimer’s disease: revising the NINCDS-ADRDA criteria. Lancet Neurol. 2007; 6:734–46. 10.1016/S1474-4422(07)70178-3 17616482

[20] McKhann G, Drachman D, Folstein M, Katzman R, Price D, Stadlan EM. Clinical diagnosis of Alzheimer’s disease: report of the NINCDS-ADRDA work group under the auspices of department of health and human services task force on Alzheimer’s disease. Neurology. 1984; 34:939–44. 10.1212/wnl.34.7.939 6610841

[21] Beach TG, Monsell SE, Phillips LE, Kukull W. Accuracy of the clinical diagnosis of Alzheimer disease at national institute on aging Alzheimer disease centers, 2005-2010. J Neuropathol Exp Neurol. 2012; 71:266–73. 10.1097/NEN.0b013e31824b211b 22437338 PMC3331862

[22] Lim A, Tsuang D, Kukull W, Nochlin D, Leverenz J, McCormick W, Bowen J, Teri L, Thompson J, Peskind ER, Raskind M, Larson EB. Clinico-neuropathological correlation of Alzheimer’s disease in a community-based case series. J Am Geriatr Soc. 1999; 47:564–69. 10.1111/j.1532-5415.1999.tb02571.x 10323650

[23] Petrovitch H, White LR, Ross GW, Steinhorn SC, Li CY, Masaki KH, Davis DG, Nelson J, Hardman J, Curb JD, Blanchette PL, Launer LJ, Yano K, Markesbery WR. Accuracy of clinical criteria for AD in the Honolulu-Asia aging study, a population-based study. Neurology. 2001; 57:226–34. 10.1212/wnl.57.2.226 11468306

[24] Barthel H, Sabri O. Clinical use and utility of amyloid imaging. J Nucl Med. 2017; 58:1711–17. 10.2967/jnumed.116.185017 28818990

[25] Murray ME, Graff-Radford NR, Ross OA, Petersen RC, Duara R, Dickson DW. Neuropathologically defined subtypes of Alzheimer’s disease with distinct clinical characteristics: a retrospective study. Lancet Neurol. 2011; 10:785–96. 10.1016/S1474-4422(11)70156-9 21802369 PMC3175379

[26] Jack CR Jr, Knopman DS, Jagust WJ, Shaw LM, Aisen PS, Weiner MW, Petersen RC, Trojanowski JQ. Hypothetical model of dynamic biomarkers of the Alzheimer’s pathological cascade. Lancet Neurol. 2010; 9:119–28. 10.1016/S1474-4422(09)70299-6 20083042 PMC2819840

[27] Tateno A, Sakayori T, Kawashima Y, Higuchi M, Suhara T, Mizumura S, Mintun MA, Skovronsky DM, Honjo K, Ishihara K, Kumita S, Suzuki H, Okubo Y. Comparison of imaging biomarkers for Alzheimer’s disease: amyloid imaging with [18F]florbetapir positron emission tomography and magnetic resonance imaging voxel-based analysis for entorhinal cortex atrophy. Int J Geriatr Psychiatry. 2015; 30:505–13. 10.1002/gps.4173 25043833

[28] Jack CR Jr, Lowe VJ, Senjem ML, Weigand SD, Kemp BJ, Shiung MM, Knopman DS, Boeve BF, Klunk WE, Mathis CA, Petersen RC. 11C PiB and structural MRI provide complementary information in imaging of Alzheimer’s disease and amnestic mild cognitive impairment. Brain. 2008; 131:665–80. 10.1093/brain/awm336 18263627 PMC2730157

[29] Chételat G, Villemagne VL, Bourgeat P, Pike KE, Jones G, Ames D, Ellis KA, Szoeke C, Martins RN, O’Keefe GJ, Salvado O, Masters CL, Rowe CC, and Australian Imaging Biomarkers and Lifestyle Research Group. Relationship between atrophy and beta-amyloid deposition in Alzheimer disease. Ann Neurol. 2010; 67:317–24. 10.1002/ana.21955 20373343

[30] Bourgeat P, Chételat G, Villemagne VL, Fripp J, Raniga P, Pike K, Acosta O, Szoeke C, Ourselin S, Ames D, Ellis KA, Martins RN, Masters CL, et al, and AIBL Research Group. Beta-amyloid burden in the temporal neocortex is related to hippocampal atrophy in elderly subjects without dementia. Neurology. 2010; 74:121–27. 10.1212/WNL.0b013e3181c918b5 20065247

[31] Dickerson BC, Bakkour A, Salat DH, Feczko E, Pacheco J, Greve DN, Grodstein F, Wright CI, Blacker D, Rosas HD, Sperling RA, Atri A, Growdon JH, et al. The cortical signature of Alzheimer’s disease: regionally specific cortical thinning relates to symptom severity in very mild to mild AD dementia and is detectable in asymptomatic amyloid-positive individuals. Cereb Cortex. 2009; 19:497–510. 10.1093/cercor/bhn113 18632739 PMC2638813

[32] Whitwell JL, Tosakulwong N, Weigand SD, Senjem ML, Lowe VJ, Gunter JL, Boeve BF, Knopman DS, Dickerson BC, Petersen RC, Jack CR Jr. Does amyloid deposition produce a specific atrophic signature in cognitively normal subjects? Neuroimage Clin. 2013; 2:249–57. 10.1016/j.nicl.2013.01.006 24179779 PMC3778266

[33] Apostolova LG, Zarow C, Biado K, Hurtz S, Boccardi M, Somme J, Honarpisheh H, Blanken AE, Brook J, Tung S, Lo D, Ng D, Alger JR, et al, and EADC-ADNI Working Group on the Harmonized Protocol for Manual Hippocampal Segmentation. Relationship between hippocampal atrophy and neuropathology markers: a 7T MRI validation study of the EADC-ADNI harmonized hippocampal segmentation protocol. Alzheimers Dement. 2015; 11:139–50. 10.1016/j.jalz.2015.01.001 25620800 PMC4348340

[34] Iizuka T, Kameyama M. Cingulate island sign on FDG-PET is associated with medial temporal lobe atrophy in dementia with lewy bodies. Ann Nucl Med. 2016; 30:421–29. 10.1007/s12149-016-1076-9 27098829

[35] Whitwell JL, Josephs KA, Murray ME, Kantarci K, Przybelski SA, Weigand SD, Vemuri P, Senjem ML, Parisi JE, Knopman DS, Boeve BF, Petersen RC, Dickson DW, Jack CR Jr. MRI correlates of neurofibrillary tangle pathology at autopsy: a voxel-based morphometry study. Neurology. 2008; 71:743–49. 10.1212/01.wnl.0000324924.91351.7d 18765650 PMC2676950

[36] Whitwell JL, Dickson DW, Murray ME, Weigand SD, Tosakulwong N, Senjem ML, Knopman DS, Boeve BF, Parisi JE, Petersen RC, Jack CR Jr, Josephs KA. Neuroimaging correlates of pathologically defined subtypes of Alzheimer’s disease: a case-control study. Lancet Neurol. 2012; 11:868–77. 10.1016/S1474-4422(12)70200-4 22951070 PMC3490201

[37] Whitwell JL, Petersen RC, Negash S, Weigand SD, Kantarci K, Ivnik RJ, Knopman DS, Boeve BF, Smith GE, Jack CR Jr. Patterns of atrophy differ among specific subtypes of mild cognitive impairment. Arch Neurol. 2007; 64:1130–38. 10.1001/archneur.64.8.1130 17698703 PMC2735186

[38] Harper L, Fumagalli GG, Barkhof F, Scheltens P, O’Brien JT, Bouwman F, Burton EJ, Rohrer JD, Fox NC, Ridgway GR, Schott JM. MRI visual rating scales in the diagnosis of dementia: evaluation in 184 post-mortem confirmed cases. Brain. 2016; 139:1211–25. 10.1093/brain/aww005 26936938 PMC4806219

[39] Sakurai K, Tokumaru AM, Shimoji K, Murayama S, Kanemaru K, Morimoto S, Aiba I, Nakagawa M, Ozawa Y, Shimohira M, Matsukawa N, Hashizume Y, Shibamoto Y. Beyond the midbrain atrophy: wide spectrum of structural MRI finding in cases of pathologically proven progressive supranuclear palsy. Neuroradiology. 2017; 59:431–43. 10.1007/s00234-017-1812-4 28386688

