# Supplementary material for: Voxel-based morphometry focusing on medial temporal lobe structures has a limited capability to detect amyloid β, an Alzheimer’s disease pathology
Source: Aging (Albany NY). 2020 Oct 5;12(19):19701–10. doi: 10.18632/aging.104012 (PMC7732322; doi:10.18632/aging.104012)
Supplement: Supplementary Figure 1 [file aging-12-104012-s001..pdf]

SUPPLEMENTARY FIGURE

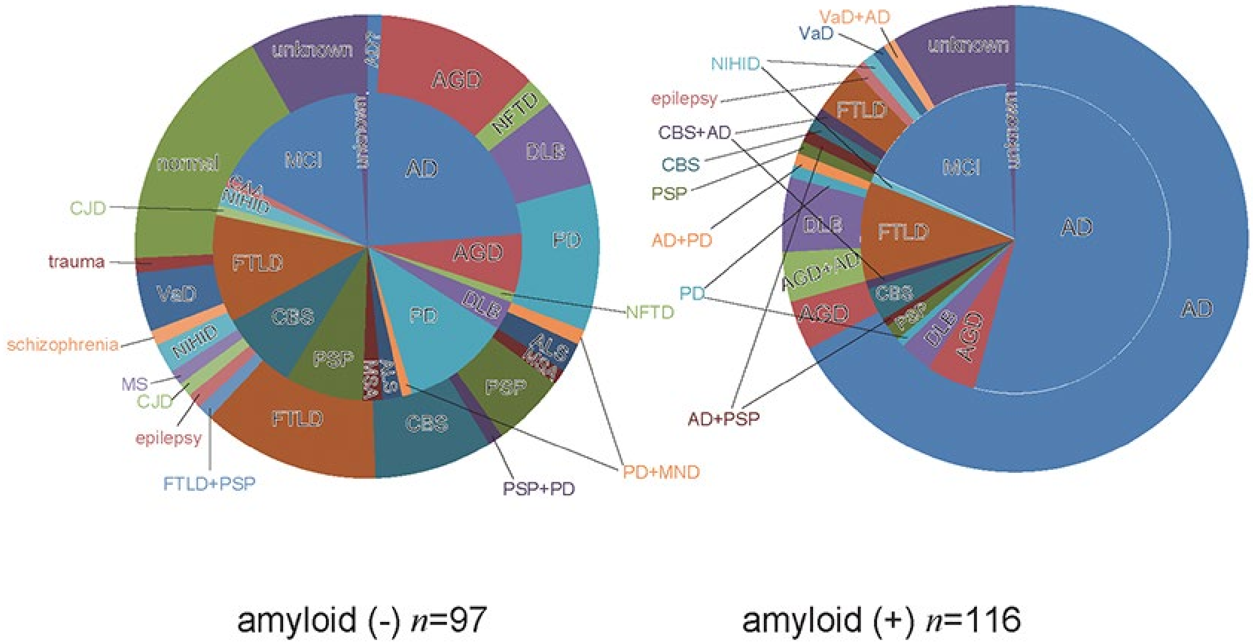

Supplementary Figure 1. Clinical diagnosis of participants. Inside: before amyloid PET; Outside: latest diagnosis.
